# Supplementary material for: Integrative Physiological, Transcriptome, and Proteome Analyses Provide Insights into the Photosynthetic Changes in Maize in a Maize–Peanut Intercropping System
Source: Plants (Basel). 2023 Dec 24;13(1):65. doi: 10.3390/plants13010065 (PMC10780508; doi:10.3390/plants13010065)
Supplement: Supplementary file 1 [file plants-13-00065-s001.zip › plants-2717693-supplementary.pdf]

Table S1. Primers and their sequences used for the quantitative real-time PCR (qRT-PCR) analysis.

Primer name	Sequence
ZmCAB1-F	CTTCGTGCAGGCTATCGTCA
ZmCAB1-S	CGTAAGCCCAGGCGTTGT
ZmBCP-F	TTACGGCAGGTCTGCTGGTA
ZmBCP-S	GTCGCTGTAGGACTGGATGGA
ZmPEPC-F	ATCCTGCACATGCTCAACCTC
ZmPEPC-S	TTAGGCACGCCCTTCCATAC
ZmPPDK-F	CCGTCAAGTCGCTAACAAAGT
ZmPPDK-S	GCTGCCCTGGGAATCTCAATC
ZmMDH-F	CTCTCCGCCTCCTCCTCTAC
ZmMDH-S	TTCCATTCCCTCCTTCCTGG
ZmrbcLBP-F	CCTGGTGCAGCAGCTAATC
ZmrbcLBP-S	ATCTACCGGCTCGAACTCA
ZmCP12-1-F	GCATCAAGCAGGCGGAGGA
ZmCP12-1-S	GGGTCGTTGGCGTTGGTCT
ZmME-F	ACGGAGAAAGAGCGGGACA
ZmME-S	GCTGCAACGGGATCTGGAA
ZmSOD-F	CATAGCCGAGGCAACCATT
ZmSOD-S	CGTAGCTGCAACTGCAACCC
ZmPOD-F	TGTCGTTGCCGTTGAGATTAC
ZmPOD-S	GTCACTGAGACCCATTTGAGC
ZmGAPDH-F	CGTCCATGCTCTCCGCTACC
ZmGAPDH-S	GCCACCTTCAGCTTCGCCTC

Table S2. Global statistics of RNA-seq results between maize monocropping and maize-peanut intercropping.

Treatments	Total reads	Total base (bp)	GC%	Q30%
T1-1	22976739	5787029038	55.62	85.71
T1-2	26424079	6655010166	57.06	85.71
T1-3	25214850	6346506801	56.46	85.24
T2-1	25709362	5192479847	57.91	87.44
T2-2	26107181	5272662740	58.31	88.25
T2-3	24704364	4989329004	58.39	87.94

Table S3. Statistical results of protein identification.

Database	Total spectra	Spectra (PSM)	Peptides	Unique peptides	Protein groups
maize	291823	51015	22092	17644	4997
